# Telotristat ethyl in carcinoid syndrome: safety and efficacy in the TELECAST phase 3 trial

**DOI:** 10.1530/ERC-17-0455

**Published:** 2018-01-09

**Authors:** Marianne Pavel, David J Gross, Marta Benavent, Petros Perros, Raj Srirajaskanthan, Richard R P Warner, Matthew H Kulke, Lowell B Anthony, Pamela L Kunz, Dieter Hörsch, Martin O Weickert, Pablo Lapuerta, Wenjun Jiang, Kenneth Kassler-Taub, Suman Wason, Rosanna Fleming, Douglas Fleming, Rocio Garcia-Carbonero

**Affiliations:** 1 Department of Gastroenterology and HepatologyCharité–Universitätsmedizin, Berlin, Germany; 2 Neuroendocrine Tumor UnitEndocrinology and Metabolism Service, Hadassah-Hebrew University Medical Center, Jerusalem, Israel; 3 Laboratorio de Oncología Molecular y Nuevas TerapiasInstituto de Biomedicina de Sevilla, Sevilla, Spain; 4 Medical Oncology DepartmentHospital Universitario Virgen del Rocio, Sevilla, Spain; 5 Department of EndocrinologyRoyal Victoria Infirmary, Newcastle Upon Tyne, UK; 6 Neuroendocrine Tumour UnitInstitute of Liver Studies, Kings College Hospital, London, UK; 7 Division of GastroenterologyIcahn School of Medicine at Mount Sinai, New York, New York, USA; 8 Medical Oncology/Solid Tumor OncologyDana-Farber Cancer Institute, Boston, Massachusetts, USA; 9 Division of Medical OncologyUniversity of Kentucky, Lexington, Kentucky, USA; 10 Department of MedicineStanford University School of Medicine, Palo Alto, California, USA; 11 Department of Gastroenterology/EndocrinologyZentralklinik Bad Berka, Bad Berka, Germany; 12 The ARDEN NET CentreENETS Centre of Excellence, University Hospitals Coventry and Warwickshire NHS Trust, Coventry, UK; 13 Lexicon PharmaceuticalsInc., The Woodlands, Texas, USA; 14 Ipsen BioscienceCambridge, Massachusetts, USA; 15 Oncology DepartmentHospital Universitario 12 de Octubre, Instituto de Investigación Sanitaria Hospital 12 de Octubre (imas12), UCM, CNIO, CIBERONC, Madrid, Spain

**Keywords:** metastatic neuroendocrine tumor, 5-HIAA, somatostatin analog, serotonin, carcinoid syndrome

## Abstract

Telotristat ethyl, a tryptophan hydroxylase inhibitor, was efficacious and well tolerated in the phase 3 TELESTAR study in patients with carcinoid syndrome (CS) experiencing ≥4 bowel movements per day (BMs/day) while on somatostatin analogs (SSAs). TELECAST, a phase 3 companion study, assessed the safety and efficacy of telotristat ethyl in patients with CS (diarrhea, flushing, abdominal pain, nausea or elevated urinary 5-hydroxyindoleacetic acid (u5-HIAA)) with <4 BMs/day on SSAs (or ≥1 symptom or ≥4 BMs/day if not on SSAs) during a 12-week double-blind treatment period followed by a 36-week open-label extension (OLE). The primary safety and efficacy endpoints were incidence of treatment-emergent adverse events (TEAEs) and percent change from baseline in 24-h u5-HIAA at week 12. Patients (*N* = 76) were randomly assigned (1:1:1) to receive placebo or telotristat ethyl 250 mg or 500 mg 3 times per day (tid); 67 continued receiving telotristat ethyl 500 mg tid during the OLE. Through week 12, TEAEs were generally mild to moderate in severity; 5 (placebo), 1 (telotristat ethyl 250 mg) and 3 (telotristat ethyl 500 mg) patients experienced serious events, and the rate of TEAEs in the OLE was comparable. At week 12, significant reductions in u5-HIAA from baseline were observed, with Hodges–Lehmann estimators of median treatment differences from placebo of −54.0% (95% confidence limits, −85.0%, −25.1%, *P* < 0.001) and −89.7% (95% confidence limits, −113.1%, −63.9%, *P* < 0.001) for telotristat ethyl 250 mg and 500 mg. These results support the safety and efficacy of telotristat ethyl when added to SSAs in patients with CS diarrhea (ClinicalTrials.gov identifier: Nbib2063659).

## Introduction

Carcinoid syndrome (CS) is a disorder that develops in up to 20% of patients with neuroendocrine tumors (NETs) and is characterized by severe diarrhea, flushing, abdominal pain, and eventually cardiac valvular complications, which can lead to heart failure (Fox & Khattar 2004, Mamikunian *et al*. 2009, National Cancer Institute 2015, Davar *et al*. 2017, Halperin *et al*. 2017, National Comprehensive Cancer Network 2017). Overproduction of serotonin by NETs results in elevated systemic levels of serotonin and can be measured by tracking the urinary metabolite 5-hydroxyindoleacetic acid (u5-HIAA). Elevated levels of serotonin occur mainly in metastatic disease and are associated with diarrhea, one of the most common symptoms of CS, and the development of carcinoid heart disease; further, high levels of u5-HIAA in patients with NETs have been associated with poor survival (Moertel 1987, van der Horst-Schrivers *et al*. 2007, Boudreaux *et al*. 2010, National Cancer Institute 2015, National Comprehensive Cancer Network 2017).

Somatostatin analogs (SSAs), introduced in the mid-1980s for the management of CS, are considered the standard of care for patients suffering from this condition (Boudreaux *et al*. 2010, Oberg & Lamberts 2016, National Comprehensive Cancer Network 2017, Pavel *et al*. 2017*b*). SSAs inhibit serotonin secretion and are an effective initial treatment for CS, but symptoms often reoccur over the course of the disease (Boudreaux *et al*. 2010, Modlin *et al*. 2010).

Telotristat ethyl, previously referred to as the hippurate salt, telotristat etiprate, is an inhibitor of tryptophan hydroxylase, the rate-limiting enzyme in serotonin biosynthesis (Liu *et al*. 2008, Kulke *et al*. 2017). The phase 3 TELESTAR study demonstrated that treatment with telotristat ethyl was generally well tolerated and was associated with significant reductions in bowel movement (BM) frequency and u5-HIAA levels in patients with CS not adequately controlled by SSA therapy (≥4 BMs per day (BMs/day) while receiving SSAs). Of note, in studies with octreotide or lanreotide conducted prior to TELESTAR, control of u5-HIAA levels had been a challenge, and dose response had not been well characterized for u5-HIAA reduction (Kvols *et al*. 1986, Rubin *et al*. 1999, Ruszniewski *et al*. 2004). Telotristat ethyl at a dosage of 250 mg 3 times per day was recently approved by the US Food and Drug Administration and the European Commission and is a category 2A recommendation in the National Comprehensive Cancer Network clinical practice guidelines for the treatment of CS diarrhea inadequately controlled by SSA therapy (European Commission 2017, FDA News Release 2017, Lexicon Pharmaceuticals 2017, National Comprehensive Cancer Network 2017).

In this international, multicenter, randomized, double-blind, placebo-controlled phase 3 companion study, TELECAST, the safety and efficacy of telotristat ethyl were assessed in patients with symptomatic CS who either had <4 BMs/day with concomitant SSA therapy (meaning that increased BM frequency was not a primary CS symptom for these patients) or who were not receiving concomitant SSA therapy (ClinicalTrials.gov identifier: Nbib2063659). The present TELECAST study aims to complement the earlier TELESTAR study by providing information on the effects of telotristat ethyl in patients who did not qualify for TELESTAR, including patients who were experiencing <4 BMs/day but had other manifestations of CS such as elevated u5-HIAA or flushing. This report constitutes the first publication of the TELECAST results, which have previously been presented in part (Pavel *et al*. 2016, 2017*a*).

## Materials and methods

### Patients

Eligible patients were ≥18 years of age and had histopathologically confirmed, well-differentiated metastatic NETs with a documented history of CS. Patients receiving SSA therapy for the treatment of CS prior to the study were required to be on stable-dose SSAs (long-acting release, depot or infusion pump) for at least 3 months prior to enrollment, be experiencing an average of <4 BMs/day and have at least 1 of the following signs or symptoms: (1) daily stool consistency ≥5 on the Bristol Stool Form scale (1 (hard lumps) to 7 (watery liquid)) for ≥50% of the days during the run-in period (indicating that the patient had diarrhea or stools that were softer than normal) (Longstreth *et al*. 2006); (2) average daily cutaneous flushing frequency of ≥2; (3) average daily rating of ≥3 for abdominal pain; (4) nausea present ≥20% of days or (5) u5-HIAA above the upper limit of normal (ULN). For patients not receiving SSA therapy, eligibility depended on having at least 1 of the above signs or symptoms or an average of ≥4 BMs/day.

Patients were excluded if they met any of the following criteria: had diarrhea attributable to any condition other than CS; were experiencing ≥4 BMs/day while on concomitant SSA therapy; showed evidence of enteric infection; had a Karnofsky performance status ≤60%; had a history of short bowel syndrome or chronic or idiopathic constipation; showed clinically important baseline elevation in liver function tests or had undergone tumor-directed therapy within 4 weeks prior to screening or hepatic embolization, radiotherapy, radiolabeled SSA therapy and/or tumor debulking within 12 weeks prior to screening. Additional exclusion criteria are described in the Supplementary methods (see section on supplementary data given at the end of this article).

### Study design and treatment

Patient baseline symptoms were established by a screening/run-in period of at least 3 weeks. Randomization was stratified by baseline u5-HIAA levels (categories of ≤ULN, >ULN, and unknown (missing at the time of randomization or uninterpretable)). Patients were randomly assigned 1:1:1 to receive oral doses of telotristat ethyl 250 mg or 500 mg 3 times per day (tid) or placebo tid during the double-blind treatment (DBT) period of 12 weeks. Patients assigned to the telotristat ethyl 500 mg group underwent a blinded titration, receiving telotristat ethyl 250 mg tid for the first 7 days. Following the DBT period, patients could opt to continue treatment with telotristat ethyl 500 mg tid in a 36-week open-label extension (OLE) period. For patients treated with placebo during the DBT period, a blinded titration with telotristat ethyl 250 mg occurred during the initial 7-day OLE period, followed by an increase to telotristat ethyl 500 mg after 7 days. Downward dose adjustment was allowed during the OLE in cases of intolerability. Rescue short-acting SSA use was allowed and unrestricted during the study, and patients continued to receive their baseline stable-dose SSA therapy.

The study protocol and amendments were approved by the institutional review board or ethics committee at each center, and the study was conducted in agreement with Good Clinical Practice guidelines and the Declaration of Helsinki. All patients provided written informed consent.

### Efficacy and safety assessments

The primary safety and efficacy endpoints were the incidence of treatment-emergent adverse events (TEAEs) and percent change from baseline in 24-h u5-HIAA levels at week 12, respectively. Adverse events (AEs) were graded in severity by the investigator as mild, moderate or severe. Event severity grading is fully described in the Supplementary methods. Depression-related AEs were prespecified as AEs of special interest (AESIs). Although not defined as such in the protocol, gastrointestinal disorder AEs and hepatic-enzyme-related AEs were also considered AESIs based on clinical experience in phase 1 and 2 studies. Planned efficacy analyses were based on the intent-to-treat population.

Key secondary efficacy endpoints included the change from baseline averaged over the 12-week DBT period for daily BM frequency, stool consistency, cutaneous flushing episodes, abdominal pain and frequency of rescue short-acting SSA treatment. Additional analyses of efficacy endpoints included durability of response to treatment (a durable response was predefined in the statistical analysis plan as a ≥30% reduction in BM frequency for ≥50% of each patient’s days on treatment in the DBT period).

Patient-reported measures were collected via an electronic patient diary (eDiary), which was identical to the one used in the TELESTAR study (Kulke *et al*. 2017) and was completed for BMs/day, number of cutaneous flushing episodes, abdominal pain/discomfort, sensation/severity of nausea, stool form/consistency, urgency to defecate, subjective global assessment of symptoms associated with CS, adequate relief of gastrointestinal symptoms of CS and need for self-administered rescue short-acting SSA therapy to treat symptoms associated with CS.

### Statistical analysis

Statistical analyses were performed using SAS statistical software (SAS Institute, Cary, NC, USA), version 9.3 or higher for the efficacy endpoints. Statistical testing used a two-sided α-level set at 0.05 in determining the significance. A blocked 2-sample Wilcoxon rank-sum statistic (stratified by baseline u5-HIAA levels) was used to evaluate treatment group differences for the primary efficacy endpoint. The nonparametric Hodges–Lehmann estimator of the median paired treatment difference was used to describe the magnitude of treatment effect. Sample size was based on percent change from baseline in 24-h u5-HIAA levels at week 12. Sixteen patients per arm would enable a power of 80% to detect a difference in u5-HIAA levels, assuming the difference between the treatment groups was 40% and the common standard deviation was 35%, leading to an effect size (mean/standard deviation) of 1.143. A target reduction of 40% was chosen to reduce the risk of capturing changes due to natural variability of u5-HIAA levels; this is higher than the threshold in prior studies with NETs in which secretory biomarker reduction of ≥30% has been used as a measure of treatment efficacy (Kulke *et al*. 2017, Yao *et al*. 2011). The evaluation of the safety endpoint was qualitative, and no statistical analyses were performed. Full statistical methods are described in the Supplementary methods.

## Results

### Patient characteristics

**Figure 1:**
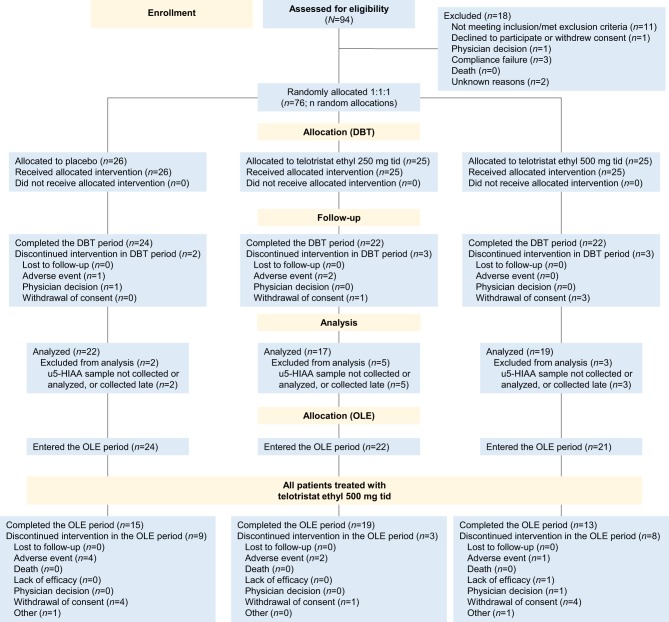
CONSORT diagram of the TELECAST clinical trial. Patient flow in the TELECAST study. DBT, double-blind treatment; OLE, open-label extension; tid, 3 times per day.

From April 2014 to April 2015, 76 patients from 11 countries were randomly assigned (1:1:1) to receive telotristat ethyl 250 mg tid, telotristat ethyl 500 mg tid or placebo tid (Fig. 1). The patient demographics and baseline characteristics are described in Table 1. They were well balanced across many of the characteristics (age, sex, ethnicity, race, weight, height, baseline body mass index, baseline u5-HIAA level); however, there were some exceptions with respect to differences in concomitant therapies. At baseline, mean BMs/day ranged from 2.2 to 2.8, whereas mean u5-HIAA levels ranged from 66.0 mg to 86.3 mg per 24 h. Across all study arms, at least 68.4% of patients had u5-HIAA levels greater than the ULN. Patients were diagnosed with a NET an average of 7.8 years prior to inclusion in this study (standard deviation (s.d.) 3.8 years; median 9.1 years; range 0.6–19.1 years). Approximately 90% (*n* = 68) of patients had medical histories of gastrointestinal disorders, most commonly diarrhea; 58% (*n* = 44) had metabolic and nutrition disorders, which included vitamin deficiencies, electrolyte abnormalities and malnutrition; and 42% (*n* = 32) of patients had a history of cardiac disorders, which included carcinoid heart disease in 9, 6 and 5 patients on placebo, telotristat ethyl 250 mg and telotristat ethyl 500 mg, respectively.

**Table 1:** Demographic and baseline characteristics of the patient population.

Patient characteristics	DBT			OLE
	Placebo (*n* = 26)	Telotristat ethyl 250 mg tid (*n* = 25)	Telotristat ethyl 500 mg tid (*n* = 25)	Telotristat ethyl 500 mg tid (*n* = 67)
Mean age, years (s.d.)	62.2 (10.3)	63.6 (12.6)	62.7 (12.0)	63.3 (11.4)
Median age (range)	65.0 (41–78)	62.0 (38–84)	63.0 (35–83)	64.0 (35–84)
Male, *n* (%)	13 (50.0)	14 (56.0)	15 (60.0)	39 (58.2)
u5-HIAA at randomization, *n* (%)^a^				
≤ULN	9 (34.6)	5 (20.0)	8 (32.0)	19 (28.4)
>ULN	17 (65.4)	18 (72.0)	17 (68.0)	46 (68.7)
Unknown	0	2 (8.0)	0	2 (3.0)
Mean u5-HIAA at baseline, mg/day (median)	82.0 (31.1)	86.3 (84.0)	66.0 (40.0)	78.2 (43.1)
Mean BM frequency, counts/day (s.d.)^b^	2.2 (0.7)	2.5 (1.2)	2.8 (1.6)	2.4 (1.1)
Averaging ≥1 BM/day at baseline, *n* (%)	25 (96.2)	24 (96.0)	24 (96.0)	64 (95.5)
Averaging ≥4 BMs/day^c^ at baseline, *n* (%)	0	1 (4.0)	4 (16.0)	3 (4.5)
Mean weekly stool consistency score (s.d.)^b^	5.0 (0.9)	5.1 (0.8)	5.3 (0.8)	5.1 (0.9)
Mean number of flushing episodes/day (s.d.)^b^	3.7 (4.1)	2.7 (3.7)	1.8 (2.2)	2.6 (3.1)
Mean weekly abdominal pain rating (s.d.)^b^	1.7 (1.7)	1.2 (1.5)	1.8 (1.7)	1.5 (1.7)
SSA therapy at study entry, *n* (%)				
Octreotide	12 (46.2)	17 (68.0)	16 (64.0)	40 (59.7)
Lanreotide	14 (53.8)	5 (20)	3 (12.0)	20 (29.9)
Unknown	0	0	1 (4.0)	1 (1.5)
Not on SSA	0	3 (12.0)	5 (20.0)	6 (9.0)

^a^Reference range: normal u5-HIAA ≤0–15 mg per 24 h (Tellez *et al*. 2013); ^b^baseline diary endpoints for placebo (*n* = 25) and OLE (*n* = 66); ^c^these patients were not currently receiving LAR/depot/infusion SSA therapy.

BM, bowel movement; DBT, double-blind treatment; OLE, open-label extension; s.d., standard deviation; SSA, somatostatin analog; tid, 3 times per day; u5-HIAA, urinary 5-hydroxyindoleacetic acid; ULN, upper limit of normal.

### Patient disposition

In this study, 26 patients received placebo, 25 patients received telotristat ethyl 250 mg tid and 25 patients received telotristat ethyl 500 mg tid in the DBT period (Fig. 1). A total of 68 patients completed the DBT period: 22 patients per telotristat ethyl group (88.0%) and 24 patients (92.3%) in the placebo group. The mean telotristat ethyl exposure time during the DBT period was 11.5 weeks (median 12.0 weeks). Mean compliance was 91.8% (s.d. 12.1%) and 92.8% (s.d. 11.8%) for the telotristat ethyl 250 mg and telotristat ethyl 500 mg groups, respectively, and 96.7% (s.d. 4.8%) for the placebo group. Reasons for withdrawal or discontinuation are shown in Fig. 1.

Of the 68 patients who completed the DBT period, 67 were enrolled in the OLE period. Patients who entered the OLE period received a mean of 30.3 additional weeks of telotristat ethyl exposure (median 36.0 weeks) from week 13 to week 48. More than half of patients who started the OLE period (47/67, 70.1%) completed the full OLE period, and compliance was 76.1%. The most common reasons for discontinuation in both the DBT and OLE periods were withdrawal of consent (*n* = 4 for DBT and *n* = 9 for OLE) and AEs (*n* = 3 for DBT and *n* = 7 for OLE).

### Primary endpoint: safety

During the DBT period, the overall incidence of any TEAEs was similar among all groups (Table 2). During the DBT period, 5 patients (19.2%) in the placebo group experienced a serious adverse event (SAE), whereas only 1 patient (4.0%) and 3 patients (12.0%) experienced a SAE in the telotristat ethyl 250 mg and telotristat ethyl 500 mg groups, respectively. TEAEs that were considered ‘treatment related’ by investigators occurred more frequently in the telotristat ethyl treatment groups compared with the placebo group. Across all 3 groups, there were no deaths during the DBT or OLE periods. Only 3 patients discontinued the study drug during the DBT period because of a TEAE: 1 patient in the placebo group (malignant neoplasm progression) and 2 patients in the telotristat ethyl 250 mg group (upper abdominal pain and diarrhea). Most TEAEs were rated as mild or moderate in severity and were most commonly gastrointestinal in nature (Tables 2 and 3).

**Table 2:** Number of patients with TEAEs reported in the DBT and OLE periods.

Category, *n* (%)	DBT			OLE
	Placebo (*n* = 26)	Telotristat ethyl 250 mg tid (*n* = 25)	Telotristat ethyl 500 mg tid (*n* = 25)	Telotristat ethyl 500 mg tid (*n* = 67)
Any TEAE	21 (80.8)	25 (100.0)	22 (88.0)	61 (91.0)
TEAE by severity^a^				
Mild	9 (34.6)	9 (36.0)	10 (40.0)	11 (16.4)
Moderate	11 (42.3)	13 (52.0)	11 (44.0)	30 (44.8)
Severe	1 (3.8)	3 (12.0)	1 (4.0)	20 (29.9)
Treatment-related TEAEs	7 (26.9)	10 (40.0)	11 (44.0)	28 (41.8)
Serious TEAEs^b^	5 (19.2)	1 (4.0)	3 (12.0)	17 (25.4)
Treatment-related serious TEAEs^c^	0	0	0	2 (3.0)
Study discontinuation due to TEAEs^d^	1 (3.8)	2 (8.0)	0	7 (10.4)
TEAE resulting in death	0	0	0	0

^a^Severity grades are defined in the Supplementary methods; patients with ≥1 TEAE in a given period are counted once at the maximum severity across all the patient’s TEAEs for that period; ^b^life-threatening AE, death, hospitalization, persistent or significant incapacity or disruption of ability to conduct normal life functions, or congenital anomaly or birth defect; ^c^treatment-related serious TEAEs during the OLE period were acute myocardial infarction and small intestinal hemorrhage (1 patient each); ^d^TEAEs leading to study discontinuation in the DBT period were upper abdominal pain, diarrhea, and tumor progression (1 patient each), and in the OLE period they were small intestinal hemorrhage, performance status decrease, elevated gamma-glutamyl transferase or hepatic enzymes, tumor progression (1 patient each), and depression (2 patients).

AE, adverse event; DBT, double-blind treatment; OLE, open-label extension; TEAE, treatment-emergent adverse event; tid, 3 times per day.

**Table 3:** Incidence of TEAEs in ≥5% of patients in any group.

System organ class preferred term, *n* (%)	DBT			OLE
	Placebo (*n* = 26)	Telotristat ethyl 250 mg tid (*n* = 25)	Telotristat ethyl 500 mg tid (*n* = 25)	Telotristat ethyl 500 mg tid (*n* = 67)
Blood and lymphatic system disorders^†^	1 (3.8)	1 (4.0)	1 (4.0)	4 (6.0)
Anemia	0	1 (4.0)	1 (4.0)	4 (6.0)
Cardiac disorders^†^	2 (7.7)	0	0	12 (17.9)
Ear and labyrinth disorders^†^	0	0	0	4 (6.0)
Gastrointestinal disorders^†^	15 (57.7)	16 (64.0)	10 (40.0)	39 (58.2)
Nausea	4 (15.4)	3 (12.0)	2 (8.0)	14 (20.9)
Abdominal pain	4 (15.4)	8 (32.0)	1 (4.0)	12 (17.9)
Diarrhea	5 (19.2)	4 (16.0)	2 (8.0)	9 (13.4)
Constipation	1 (3.8)	4 (16.0)	3 (12.0)	8 (11.9)
Vomiting	1 (3.8)	1 (4.0)	1 (4.0)	7 (10.4)
Abdominal pain upper	3 (11.5)	1 (4.0)	2 (8.0)	5 (7.5)
Abdominal distension	0	3 (12.0)	1 (4.0)	4 (6.0)
Dyspepsia	2 (7.7)	2 (8.0)	0	0
General disorders and administration site conditions^†^	6 (23.1)	8 (32.0)	4 (16.0)	24 (35.8)
Fatigue	2 (7.7)	3 (12.0)	2 (8.0)	7 (10.4)
Pyrexia	0	3 (12.0)	0	6 (9.0)
Asthenia	2 (7.7)	1 (4.0)	0	7 (10.4)
Peripheral edema	0	2 (8.0)	1 (4.0)	5 (7.5)
Infections and infestations^†^	5 (19.2)	8 (32.0)	5 (20.0)	17 (25.4)
Urinary tract infection	0	3 (12.0)	0	3 (4.5)
Influenza	0	0	2 (8.0)	1 (1.5)
Nasopharyngitis	1 (3.8)	1 (4.0)	0	5 (7.5)
Injury, poisoning, and procedural complications^†,a^	1 (3.8)	0	1 (4.0)	9 (13.4)
Investigations^†,b^	3 (11.5)	3 (12.0)	2 (8.0)	18 (26.9)
Gamma-glutamyl transferase increase	0	1 (4.0)	1 (4.0)	4 (6.0)
Weight decreased	0	1 (4.0)	0	4 (6.0)
Metabolism and nutrition disorders^†^	0	1 (4.0)	2 (8.0)	14 (20.9)
Decreased appetite	0	0	2 (8.0)	6 (9.0)
Musculoskeletal and connective tissue disorders^†,b^	4 (15.4)	1 (4.0)	5 (20.0)	22 (32.8)
Myalgia	2 (7.7)	1 (4.0)	1 (4.0)	2 (3.0)
Musculoskeletal pain	0	0	2 (8.0)	2 (3.0)
Arthralgia	1 (3.8)	0	0	4 (6.0)
Back pain	1 (3.8)	0	0	5 (7.5)
Neoplasms benign, malignant, and unspecified (including cysts and polyps)^†^	1 (3.8)	1 (4.0)	4 (16.0)	5 (7.5)
Nervous system disorders^†^	5 (19.2)	2 (8.0)	3 (12.0)	16 (23.9)
Dizziness	3 (11.5)	0	2 (8.0)	3 (4.5)
Headache	1 (3.8)	0	1 (4.0)	5 (7.5)
Presyncope	0	1 (4.0)	0	5 (7.5)
Psychiatric disorders^†,b^	2 (7.7)	2 (8.0)	2 (8.0)	16 (23.9)
Depression	0	0	1 (4.0)	8 (11.9)
Depressed mood	2 (7.7)	1 (4.0)	0	2 (3.0)
Renal and urinary disorders^†^	1 (3.8)	3 (12.0)	0	8 (11.9)
Respiratory, thoracic, and mediastinal disorders^†^	3 (11.5)	3 (12.0)	1 (4.0)	13 (19.4)
Dyspnea	2 (7.7)	1 (4.0)	0	4 (6.0)
Oropharyngeal pain	0	2 (8.0)	0	1 (1.5)
Cough	0	1 (4.0)	0	4 (6.0)
Skin and subcutaneous tissue disorders^†^	1 (3.8)	6 (24.0)	3 (12.0)	10 (14.9)
Night sweats	0	2 (8.0)	1 (4.0)	1 (1.5)
Surgical and medical procedures^†^	1 (3.8)	0	0	8 (11.9)
Vascular disorders^†^	4 (15.4)	5 (20.0)	2 (8.0)	15 (22.4)
Flushing	2 (7.7)	3 (12.0)	0	9 (13.4)

^†^Subcategories experienced by <5% of patients are not included; ^a^preferred terms included for injury, poisoning, and procedural complications are listed in the Supplementary methods. Each of these categories was experienced by <5% of patients; ^b^preferred terms also included for investigations, musculoskeletal and connective tissue disorders, and psychiatric disorders are listed in the Supplementary methods. Each of these categories was experienced by <5% of patients.

DBT, double-blind treatment; OLE, open-label extension; TEAE, treatment-emergent adverse event; tid, 3 times per day.

During the OLE, the types of safety-related events, incidences of overall TEAEs and incidences of TEAEs leading to study discontinuation were similar to those in the DBT period, considering the increased exposure to telotristat ethyl. Crossover from placebo or telotristat ethyl 250 mg to telotristat ethyl 500 mg in the OLE period did not affect the incidence of TEAEs. During the OLE period, only 2 patients required downward dose adjustment to 250 mg telotristat ethyl tid. TEAEs were generally mild or moderate in intensity, with the most frequently reported incidence being gastrointestinal in nature, specifically nausea. Four patients had TEAEs that were reported as both severe and treatment related by the investigator (Table 2; 1 patient each: acute myocardial infarction, constipation, a small intestinal hemorrhage and an increase in gamma-glutamyl transferase (GGT)).

AEs of special interest included depression, gastrointestinal disorders and hepatic enzyme elevations (Table 4) based on mechanism of action or previous clinical experience with telotristat ethyl (Liu *et al*. 2008, Kulke *et al*. 2017). There was no increase in depression-related AEs on telotristat ethyl compared with placebo, and no severe or serious cases in either the DBT or OLE period. Two patients suffered from AEs related to depression (both of moderate severity), leading to study discontinuation in the OLE period; both patients had underlying depression and were receiving antidepressant therapy at baseline.

**Table 4:** Adverse events of special interest.

AESI category preferred term, *n* (%)		DBT			OLE
		Placebo tid (*n* = 26)	Telotristat ethyl 250 mg tid (*n* = 25)	Telotristat ethyl 500 mg tid (*n* = 25)	Telotristat ethyl 500 mg tid (*n* = 67)
**Gastrointestinal-related TEAE**					
Abdominal pain	Total	4 (15.4)	8 (32.0)	1 (4.0)	12 (17.9)
	Mild	1 (3.8)	3 (12.0)	0	5 (7.5)
	Moderate	3 (11.5)	3 (12.0)	0	6 (9.0)
	Severe	0	2 (8.0)	1 (4.0)	1 (1.5)
Diarrhea	Total	5 (19.2)	4 (16.0)	2 (8.0)	9 (13.4)
	Mild	2 (7.7)	2 (8.0)	1 (4.0)	3 (4.5)
	Moderate	3 (11.5)	2 (8.0)	1 (4.0)	4 (6.0)
	Severe	0	0	0	2 (3.0)
Nausea	Total	4 (15.4)	3 (12.0)	2 (8.0)	14 (20.9)
	Mild	4 (15.4)	2 (8.0)	2 (8.0)	8 (11.9)
	Moderate	0	1 (4.0)	0	5 (7.5)
	Severe	0	0	0	1 (1.5)
Constipation	Total	1 (3.8)	4 (16.0)	3 (12.0)	8 (11.9)
	Mild	0	3 (12.0)	3 (12.0)	5 (7.5)
	Moderate	1 (3.8)	1 (4.0)	0	2 (3.0)
	Severe	0	0	0	1 (1.5)
Abdominal pain upper	Total	3 (11.5)	1 (4.0)	2 (8.0)	5 (7.5)
	Mild	2 (7.7)	0	2 (8.0)	3 (4.5)
	Moderate	1 (3.8)	1 (4.0)	0	2 (3.0)
	Severe	0	0	0	0
Abdominal distension	Total	0	3 (12.0)	1 (4.0)	4 (6.0)
	Mild	0	2 (8.0)	1 (4.0)	4 (6.0)
	Moderate	0	1 (4.0)	0	0
	Severe	0	0	0	0
Abdominal discomfort	Total	1 (3.8)	0	1 (4.0)	1 (1.5)
	Mild	1 (3.8)	0	1 (4.0)	1 (1.5)
	Moderate	0	0	0	0
	Severe	0	0	0	0
Flatulence	Total	0	1 (4.0)	1 (4.0)	2 (3.0)
	Mild	0	1 (4.0)	1 (4.0)	1 (1.5)
	Moderate	0	0	0	1 (1.5)
	Severe	0	0	0	0
Abdominal tenderness	Total	0	0	1 (4.0)	0
	Mild	0	0	1 (4.0)	0
	Moderate	0	0	0	0
	Severe	0	0	0	0
Frequent bowel movements	Total	0	1 (4.0)	0	0
	Mild	0	0	0	0
	Moderate	0	1 (4.0)	0	0
	Severe	0	0	0	0
Abdominal pain lower	Total	0	0	0	1 (1.5)
	Mild	0	0	0	0
	Moderate	0	0	0	1 (1.5)
	Severe	0	0	0	0
Gastrointestinal pain	Total	0	0	0	1 (1.5)
	Mild	0	0	0	1 (1.5)
	Moderate	0	0	0	0
	Severe	0	0	0	0
Vomiting	Total	1 (3.8)	1 (4.0)	1 (4.0)	7 (10.4)
	Mild	0	1 (4.0)	0	4 (6.0)
	Moderate	1 (3.8)	0	1 (4.0)	2 (3.0)
	Severe	0	0	0	1 (1.5)
**Depression-related TEAE**					
Depressed mood	Total	2 (7.7)	1 (4.0)	0	2 (3.0)
	Mild	2 (7.7)	1 (4.0)	0	2 (3.0)
	Moderate	0	0	0	0
	Severe	0	0	0	0
Decreased interest	Total	1 (3.8)	0	0	0
	Mild	1 (3.8)	0	0	0
	Moderate	0	0	0	0
	Severe	0	0	0	0
Depression	Total	0	0	1 (4.0)	8 (11.9)
	Mild	0	0	0	4 (6.0)
	Moderate	0	0	1 (4.0)	4 (6.0)
	Severe	0	0	0	0
**Hepatic-enzyme-related TEAE**					
Elevated gamma-glutamyl transferase	Total	0	1 (4.0)	1 (4.0)	4 (6.0)
	Mild	0	1 (4.0)	0	1 (1.5)
	Moderate	0	0	1 (4.0)	1 (1.5)
	Severe	0	0	0	2 (3.0)
Elevated alanine aminotransferase	Total	0	1 (4.0)	0	3 (4.5)
	Mild	0	1 (4.0)	0	2 (3.0)
	Moderate	0	0	0	0
	Severe	0	0	0	1 (1.5)
Liver function test abnormal	Total	0	0	1 (4.0)	1 (1.5)
	Mild	0	0	1 (4.0)	0
	Moderate	0	0	0	1 (1.5)
	Severe	0	0	0	0
Elevated aspartate aminotransferase	Total	0	0	0	3 (4.5)
	Mild	0	0	0	2 (3.0)
	Moderate	0	0	0	0
	Severe	0	0	0	1 (1.5)
Blood alkaline phosphatase increased	Total	0	0	0	2 (3.0)
	Mild	0	0	0	1 (1.5)
	Moderate	0	0	0	1 (1.5)
	Severe	0	0	0	0
Hepatic enzyme increased	Total	0	0	0	2 (3.0)
	Mild	0	0	0	1 (1.5)
	Moderate	0	0	0	1 (1.5)
	Severe	0	0	0	0

AESI, adverse event of special interest; DBT, double-blind treatment; OLE, open-label extension; TEAE, treatment-emergent adverse event; tid, 3 times per day.

AEs of special interest related to hepatic enzyme abnormalities were mostly mild or moderate in severity. Two events led to study discontinuation during the OLE period. One patient experienced elevated alanine aminotransferase (ALT), alkaline phosphatase (ALP), aspartate aminotransferase (AST) and GGT. Telotristat ethyl was discontinued, and the patient’s hepatic enzymes normalized within 3 months. Another patient had an underlying condition of elevated GGT levels at baseline and throughout the course of the study. A follow-up visit during the OLE period found that GGT levels had increased approximately 6-fold above baseline, and telotristat ethyl was discontinued. During the DBT period, 2 patients (1 in each telotristat ethyl group) experienced an increase in GGT, 1 patient in the telotristat ethyl 250 mg group experienced an increase in ALT and 1 patient in the telotristat ethyl 500 mg group experienced an abnormal liver function test; however, there were no associated increases in bilirubin.

Gastrointestinal disorders (diarrhea, nausea, constipation, abdominal pain, abdominal distension, dyspepsia, vomiting and abdominal discomfort) were frequent TEAEs for all groups in both the DBT and OLE periods; however, there was no dose-dependent or drug-dependent relationship between telotristat ethyl and gastrointestinal symptoms. The most frequent severe TEAE observed in the DBT period (3 patients), abdominal pain, was seen in only 1 patient during the OLE period. Constipation was reported more often on telotristat ethyl than placebo. Constipation was rated as mild to moderate in 7 of the 8 patients reporting this event during the OLE period. Nausea occurred with similar frequencies among patients treated with placebo and telotristat ethyl during the DBT period and did not result in SAEs or treatment discontinuation.

There were no significant changes in vital signs (other than weight), electrocardiogram measurements or physical examination findings during the DBT period. With respect to weight, 2 (8.7%), 4 (17.4%) and 5 (20.8%) patients in the placebo, telotristat ethyl 250 mg and the telotristat ethyl 500 mg groups, respectively, had at least a 3% gain in body weight during the DBT period. The 3 patients with electrocardiogram changes outside of the normal range were on placebo, and 2 had underlying histories of cardiac disorders. Electrocardiogram measures included heart rate, PR duration, QRS duration and QT interval and are summarized in Supplementary Table 1.

### Primary endpoint: efficacy

**Figure 2:**
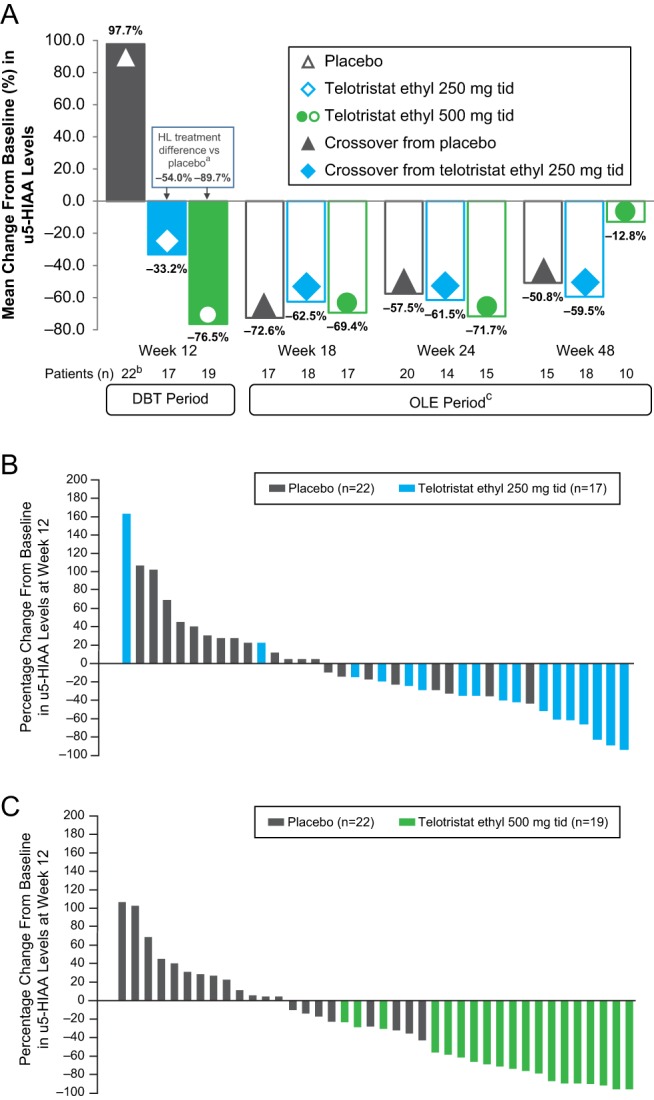
Changes in u5-HIAA levels from baseline. (A) The Hodges–Lehmann (HL) estimator, a nonparametric measure used to describe the magnitude of treatment effect, was assessed at week 12 (^a^*P* < 0.001). ^b^Data include 1 patient who experienced a 1864.5% increase from baseline. ^c^The colors and shapes in each bar represent the assigned treatment group during the double-blind treatment (DBT) period; clear bars with filled shapes indicate treatment of these patients with telotristat ethyl 500 mg 3 times per day (tid) during the open-label extension (OLE) period. (B) The distribution of individual patient responses to treatment with telotristat ethyl 250 mg tid or placebo, as percent change from baseline at week 12. One patient treated with placebo had a 1864.5% change from baseline and is not included in the figure. (C) The distribution of individual patient responses to treatment with telotristat ethyl 500 mg tid or placebo, as percent change from baseline at week 12. One patient treated with placebo had a 1864.5% change from baseline and is not included in the figure. u5-HIAA, urinary 5-hydroxyindoleacetic acid.

A statistically significant reduction from baseline in u5-HIAA levels was observed at week 12 for both the telotristat ethyl 250 mg and telotristat ethyl 500 mg groups compared with the placebo group (*P* < 0.001 for each comparison) and continued through the OLE period for the majority of patients (Fig. 2). Baseline and week 12 values were available for 17, 19 and 22 patients in the telotristat ethyl 250 mg, telotristat ethyl 500 mg and placebo groups, respectively. Ten patients who completed the DBT period were excluded from the primary efficacy analysis because of deviations related to u5-HIAA sample collection or analysis. At week 12, significant reductions in u5-HIAA levels from baseline were observed with Hodges–Lehmann estimator of treatment differences from placebo of −54.0% (95% confidence limits (CL) −85.0%, −25.1%) for the 250 mg telotristat ethyl group and −89.7% (95% CL −113.1%, −63.9%) for the 500 mg telotristat ethyl group (*P* < 0.001 for both vs placebo). Additionally, more patients in the telotristat ethyl groups demonstrated a reduction in u5-HIAA levels at week 12 compared with the placebo group (15 of 17 patients for telotristat ethyl 250 mg, 19 of 19 patients for telotristat ethyl 500 mg and 8 of 22 patients for placebo).

### Additional efficacy endpoints

**Figure 3:**
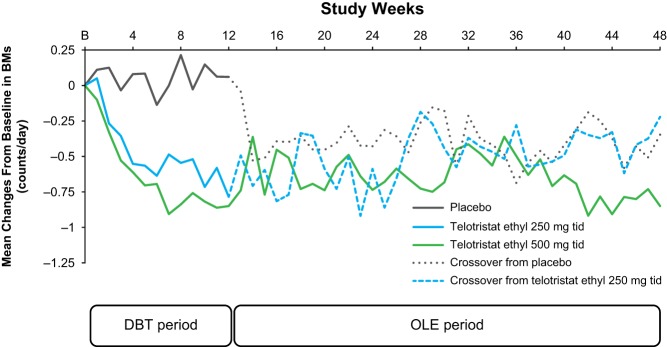
Mean changes from baseline (B) in frequency of bowel movements per day. The change in mean daily bowel movement (BM) frequency was assessed weekly over the double-blind treatment (DBT) and open-label extension (OLE) periods of the study. The dotted lines indicate the crossover of patients from either placebo or telotristat ethyl 250 mg 3 times per day (tid) to telotristat ethyl 500 mg tid in the OLE period.

Treatment with telotristat ethyl at either dosage was also associated with statistically significant reductions in BMs/day averaged over the 12-week DBT period compared with placebo (*P* = 0.004 for telotristat ethyl 250 mg and *P* < 0.001 for telotristat ethyl 500 mg, Table 5; percent change in BM frequency from baseline per week is shown in Fig. 3). The Hodges–Lehmann estimator of treatment difference from placebo for patients receiving telotristat ethyl was −0.45 for the telotristat ethyl 250 mg group and −0.54 for the telotristat ethyl 500 mg group. Durable responses (predefined in the statistical analysis plan as responders with ≥30% reduction in daily number of BMs for ≥50% of the time over the DBT period) were observed only in patients on telotristat ethyl. Ten patients in each telotristat ethyl group (40.0%) were classified as durable responders, whereas none of the patients who received placebo were classified as durable responders (treatment difference in responder rate of 0.40; 95% CL 0.17, 0.63; *P* = 0.001). During the DBT period, a mean reduction in BM frequency by approximately 1 BM/day was also observed in the small number (*n* = 8) of telotristat ethyl–treated patients who were not on concurrent SSA therapy. The mean reduction in BMs/day observed in the DBT period for telotristat ethyl–treated patients continued through the OLE period.

**Table 5:** Secondary endpoints of the double-blind treatment period.

Variable	Placebo (*n* = 25)	Telotristat ethyl					
		250 mg tid (*n* = 25)			500 mg tid (*n* = 25)		
	Mean (s.d.)	Mean (s.d.)	*P*	95% CL	Mean (s.d.)	*P*	95% CL
**BM frequency**							
Change from baseline in BM frequency averaged over 12 weeks, counts/day	0.05 (0.33)	−0.45 (0.69)	−	−	−0.60 (0.72)	−	−
Arithmetic mean treatment difference	−	−0.50	−	−0.81 to −0.19	−0.65	−	−0.96 to −0.33
Hodges–Lehmann estimator	−	−0.45	0.004	−0.72 to −0.17	−0.54	<0.001	−0.79 to −0.25
**Stool consistency**							
Change from baseline in stool consistency averaged over 12 weeks (Bristol Stool Form scale)	0.01 (0.41)	−0.20 (0.70)	–	–	−0.60 (0.86)	–	–
Arithmetic mean treatment difference	−	−0.20	–	−0.53 to 0.13	−0.60	–	−0.99 to −0.22
Hodges–Lehmann estimator	−	−0.20	0.09	−0.45 to 0.02	−0.39	0.009	−0.82 to −0.12
**Flushing**							
Change from baseline in daily cutaneous flushing episodes averaged over 12 weeks, counts/day	−0.33 (1.22)	−0.06 (0.98)	–	–	0.11 (2.10)	–	–
Arithmetic mean treatment difference	−	0.27	–	−0.36 to 0.90	0.45	–	−0.53 to 1.42
Hodges–Lehmann estimator	−	0.11	0.67	−0.17 to 0.61	0.02	0.58	−0.28 to 0.62
**Abdominal pain**							
Change from baseline in abdominal pain averaged over 12 weeks, 11-point numeric rating scale	−0.06 (0.78)	−0.23 (0.97)	–	–	0.03 (0.77)	–	–
Arithmetic mean treatment difference	−	−0.17	–	−0.67 to 0.33	0.09	–	−0.35 to 0.53
Hodges–Lehmann estimator	−	0.06	0.61	−0.42 to 0.33	0.14	0.66	−0.39 to 0.51
**Rescue short-acting SSA use**							
Change from baseline in rescue short-acting SSA use averaged over 12 weeks, counts/day	−0.01 (0.14)	−0.07 (0.35)	–	–	0.01 (0.10)	–	–
Arithmetic mean treatment difference	−	−0.05	–	−0.20 to 0.10	0.02	–	−0.05 to 0.09
Hodges–Lehmann estimator	−	0.00	0.45	0.00–0.00	0.000	0.98	0.00–0.00

BM, bowel movement; CL, confidence limits; s.d., standard deviation; SSA, somatostatin analog; tid, 3 times per day.

Telotristat ethyl 500 mg had a statistically significant (*P* = 0.009) effect on stool form/consistency, as measured by the Bristol Stool Form scale, compared with placebo during the DBT period. From the Hodges–Lehmann estimator, there was a difference of −0.39 (95% CL −0.82, −0.12). There was no statistically significant difference between the telotristat ethyl 250 mg group and the placebo group (*P* = 0.09), with the Hodges–Lehmann estimator at −0.20 (95% CL −0.45, 0.02).

During the DBT period, there were no statistically significant changes in cutaneous flushing, abdominal pain or frequency of rescue SSA therapy to treat CS symptoms. There was also no trend in the change from baseline in the number of rescue short-acting SSAs used to treat bowel-related CS symptoms across all time points in the DBT period, with mean changes of −0.01 counts per day (s.d. 0.14), −0.07 (s.d. 0.35) and 0.01 (s.d. 0.10) for placebo, telotristat ethyl 250 mg and telotristat ethyl 500 mg, respectively.

## Discussion

There is a substantial need for safe and effective treatment to supplement the current standard of care of SSA therapy for the management of CS including diarrhea. Toward this end, this study met both primary safety and efficacy endpoints in the 12-week DBT period comparing 2 dosing regimens of telotristat ethyl vs placebo. During both the 12-week DBT period and the OLE period of up to 36 weeks, treatment with telotristat ethyl was well tolerated, as indicated by the rarity of SAEs or discontinuations due to AEs. Overall, TEAEs and discontinuations due to TEAEs were similar among all treatment groups.

Notably, patients in this study had considerable comorbidities associated with metastatic NETs, such as gastrointestinal symptoms (most commonly diarrhea), metabolic and nutrition disorders and cardiac disorders, and patients were an average of 7.8 years since their diagnosis of a NET at inclusion in this study. Despite these comorbidities and the length of follow-up, there were no deaths during the DBT or OLE period, and more SAEs occurred in the placebo group compared with the telotristat ethyl groups during the DBT period.

The results for depression-related AEs were favorable compared with those reported in the phase 3 TELESTAR study. Previously, there has been a concern about depression with telotristat ethyl because of the known role of serotonin in depression; the pharmacologic profiles of earlier tryptophan hydroxylase inhibitors, which crossed the blood–brain barrier; and the reported numerically higher incidence of depression on telotristat ethyl 500 mg tid than on telotristat ethyl 250 mg tid or placebo during the TELESTAR study (Kulke *et al*. 2017). As reported here in the TELECAST study, during the DBT period, depression-related AEs were reported more often on placebo (2 patients) compared with either dosage of telotristat ethyl (1 patient in each treatment group) during the DBT period. During the OLE period, depression led to discontinuation of therapy in 2 patients. Overall, in TELECAST, there was no imbalance in depression-related AEs during the DBT period. The slightly higher incidence of depression observed with telotristat ethyl 500 mg tid in the OLE period (11.9%) compared with the same dosage during the DBT period (4%) suggests that the emergence of depression could be time dependent. However, telotristat ethyl is unlikely to cross the blood–brain barrier (Liu *et al*. 2008, Lapuerta *et al*. 2015), and the depression observed in TELESTAR and TELECAST might be related to the underlying disease or other causes; nevertheless, patients should be monitored for the occurrence of depression or depressed mood.

Consistent with the results of the TELESTAR study, elevations in hepatic enzymes appeared to be associated with telotristat ethyl (Kulke *et al*. 2017). Elevated hepatic enzymes (primarily GGT, with some elevations in ALT, AST and ALP) were observed in some patients, but there were no reported cases with clinically significant outcomes and no cases of coincident substantial increases in bilirubin.

Constipation also appeared to be drug related, and this is consistent with the mechanism of action of telotristat ethyl. In this study, most constipation-related AEs were mild to moderate in severity and resulted in no discontinuations of the study drug. However, symptoms of severe constipation or severe, persistent or worsening abdominal pain should be watched for, and if observed, telotristat ethyl should be discontinued to minimize the risk of possible complications.

A significant (*P* < 0.001) reduction in u5-HIAA levels was observed compared with baseline in patients given telotristat ethyl vs placebo during the 12-week DBT period. This reduction persisted during the OLE period. Elevated levels of u5-HIAA in patients with CS have been associated with valvular heart disease, mesenteric fibrosis and poor long-term survival (van der Horst-Schrivers *et al*. 2007, Druce *et al*. 2009, Davar *et al*. 2017). Telotristat ethyl also significantly reduced BM frequency compared with placebo and produced a durable reduction in BM frequency in 40% of patients, similar to the results of the TELESTAR study (Kulke *et al*. 2017). BM frequency for patients not on SSA therapy also improved; however, the small number of patients not on SSAs and the lack of patients in this category who were randomly assigned to receive placebo limit definitive conclusions. The weight increase observed in some patients in the telotristat ethyl groups could be the result of diarrhea control leading to better nutritional status.

The effects of telotristat ethyl on u5-HIAA and the BM symptoms associated with production of serotonin by NETs are consistent with results from previous studies and with telotristat ethyl’s selective inhibition of intratumoral serotonin production (Kulke *et al*. 2017). Whereas the phase 3 TELESTAR study demonstrated the effect of telotristat ethyl in patients with CS diarrhea with ≥4 BMs/day, this study showed a statistically significant effect on u5-HIAA levels and BMs/day in patients with less frequent BMs and elevated u5-HIAA.

In conclusion, treatment with telotristat ethyl for up to 48 weeks had a favorable safety profile, was well tolerated and was associated with sustained reductions in u5-HIAA and BM frequency. Data from this study and from the previous phase 3 TELESTAR study support the use of telotristat ethyl as an addition to SSAs in patients with CS diarrhea.

## Supplementary Material

Supplementary Material 1

## Declaration of interest

M P reports personal fees from Lexicon Pharmaceuticals, Inc., Ipsen Pharmaceuticals, Inc., and Novartis Pharmaceuticals, Inc., outside the submitted work. P P reports payments to Newcastle upon Tyne Hospitals NHS Foundation Trust from Lexicon Pharmaceuticals, Inc., to cover research costs for recruiting and conducting the study. R S reports grants from Ipsen Pharmaceuticals, Inc., and Novartis Pharmaceuticals, Inc., outside of the submitted work. R R P W reports clinical trial support from Lexicon Pharmaceuticals, Inc., during the study. P L K reports grants and personal fees from Lexicon Pharmaceuticals, Inc., during the study, along with grants from Advanced Accelerator Applications, Dicerna, Esanex, Genentech, Merck, Oxigene, and Incyte and grants and personal fees from Ipsen Pharmaceuticals, Inc, and Novartis Pharmaceuticals, Inc., outside of the submitted work. D H reports personal fees from Lexicon Pharmaceuticals, Inc., and personal fees and grants from Ipsen Pharmaceuticals, Inc., during the course of the study, along with personal fees from Lexicon Pharmaceuticals, Inc., grants and personal fees from Ipsen Pharmaceuticals, Inc., Novartis Pharmaceuticals, Inc. and Pfizer Pharmaceuticals, Inc., outside of the submitted work. MOW reports personal fees from Ipsen Pharmaceuticals, Inc., and Novartis Pharmaceuticals, Inc., and grants from Pfizer outside the submitted work. P L, W J, K K-T, S W and R F are employees of Lexicon Pharmaceuticals, Inc., which may include compensation that includes stock in Lexicon Pharmaceuticals, Inc. D J G, M B, L B A and R G-C have nothing to disclose.

## Funding

This work was supported by Lexicon Pharmaceuticals, Inc., The Woodlands, TX, USA. Employees of the company were involved in the study design; the collection, analysis and interpretation of data; the writing and review of the manuscript; and the decision to submit for publication.

## Author contribution statement

Conception and design: P L, D F, M P, M H K, L B A. Provision of study materials or patients: M P, D J G, M B, P P, R S, R R P W, M H K, L B A, P L K, D H, M O W, R G-C. Collection and assembly of data: S W, R F, K K-T, M O W. Data analysis and interpretation: R G-C, S W, P P, D F, W J, R F, K K-T, M P, MH K, L B A. Manuscript writing: All authors. Final approval of manuscript: All authors. Accountable for all aspects of the work: All authors.
