# Supplementary material for: Telotristat ethyl in carcinoid syndrome: safety and efficacy in the TELECAST phase 3 trial
Source: Endocr Relat Cancer. 2018 Jan 9;25(3):309–22. doi: 10.1530/ERC-17-0455 (PMC5811631; doi:10.1530/ERC-17-0455)
Supplement: Supplementary Material 1 [file erc-25-309-s001.pdf]

Telotristat Ethyl in Carcinoid Syndrome: Safety and Efficacy in the TELECAST Phase 3 Trial

Authors: Marianne Pavel[1,2], David J. Gross[3], Marta Benavent[4,5], Petros Perros[6], Raj Srirajaskanthan[7], Richard R. P. Warner[8], Matthew H. Kulke[9], Lowell B. Anthony[10], Pamela L. Kunz[11], Dieter Hörsch[12], Martin O. Weickert[13], Pablo Lapuerta[14], Wenjun Jiang[14], Kenneth Kassler-Taub[14], Suman Wason[14], Rosanna Fleming[14], Douglas Fleming[15,16], Rocio Garcia-Carbonero[17]

[1]Department of Gastroenterology and Hepatology, Charité–Universitätsmedizin, Berlin, Germany

[2]Current affiliation: Department of Medicine 1, Division of Endocrinology, Friedrich-Alexander-Universität Erlangen-Nürnberg, Erlangen, Germany

[3]Neuroendocrine Tumor Unit, Endocrinology and Metabolism Service, Hadassah-Hebrew University Medical Center, Jerusalem, Israel

[4]Laboratorio de Oncología Molecular y Nuevas Terapias, Instituto de Biomedicina de Sevilla, Sevilla, Spain

[5]Medical Oncology Department, Hospital Universitario Virgen del Rocio, Sevilla, Spain

[6]Department of Endocrinology, Royal Victoria Infirmary, Newcastle Upon Tyne, UK

[7]Neuroendocrine Tumour Unit, Institute of Liver Studies, Kings College Hospital, London, UK

[8]Division of Gastroenterology, Icahn School of Medicine at Mount Sinai, New York, New York, USA

[9]Medical Oncology/Solid Tumor Oncology, Dana-Farber Cancer Institute, Boston, Massachusetts, USA

[10]Division of Medical Oncology, University of Kentucky, Lexington, Kentucky, USA

[11]Department of Medicine, Stanford University School of Medicine, Palo Alto, California, USA

[12]Department of Gastroenterology/Endocrinology, Zentralklinik Bad Berka, Bad Berka, Germany

[13]The ARDEN NET Centre, ENETS Centre of Excellence, University Hospitals Coventry and Warwickshire NHS Trust, Coventry, UK

[14]Lexicon Pharmaceuticals, Inc., The Woodlands, Texas, USA

[15]Ipsen Bioscience, Cambridge, Massachusetts, USA

[16]Current affiliation: Bristol-Myers Squibb, Princeton, New Jersey, USA

[17]Oncology Department, Hospital Universitario 12 de Octubre, Instituto de Investigación Sanitaria Hospital 12 de Octubre (imas12), UCM, CNIO, CIBERONC, Madrid, Spain

Supplementary Methods

Additional exclusion criteria

Patients were also excluded if they met any of the following criteria:

1) Presence of diarrhea attributed to any condition(s) other than carcinoid syndrome, including, but not limited to, fat malabsorption or bile acid malabsorption
2) Presence of > 12 watery bowel movements (BMs) per day associated with volume contraction, dehydration, or hypotension compatible with a “pancreatic cholera”-type clinical syndrome, as judged by the Investigator

3) Positive stool examination for enteric pathogens, pathogenic ova or parasites, or *Clostridium difficile* at screening
4) Karnofsky performance status ≤60%
5) Clinical laboratory values for hematology (at screening):
    a. Absolute neutrophil count ≤1500 cells/mm^3
    b. Platelets ≤75,000 cells/mm^3
    c. Hemoglobin ≤9 g/dL or ≤8 g/dL for males or females, respectively
6) Hepatic laboratory values (at screening) for:
    a. Aspartate aminotransferase (AST) or alanine aminotransferase (ALT):
        i. ≥5.5 x upper limit of normal (ULN) if the patient had a documented history of hepatic metastases
        ii. ≥2.5 x ULN if there was no documented history of hepatic metastases
    b. Total bilirubin >1.5 x ULN (unless patient had documented history of Gilbert's syndrome)
    c. Alkaline phosphatase (ALP) ≥5 x ULN only if bilirubin was >ULN (there was no upper limit for ALP if total bilirubin ≤ULN)
7) Serum creatinine ≥1.5 x ULN
8) Treatment with any tumor-directed therapy including, but not limited to: interferon, chemotherapy, mammalian target of rapamycin inhibitors <4 weeks before screening; or hepatic embolization, radiotherapy, radiolabeled somatostatin analog, and/or tumor debulking <12 weeks before screening
9) A history of short bowel syndrome

10) Current complaints of constipation or history of chronic or idiopathic constipation within 2 years before screening
11) Life expectancy ≤12 months from screening visit
12) Cardiac arrhythmia, bradycardia, or tachycardia that would compromise patient safety or study outcome

Additional safety assessments

Investigators evaluated each adverse event and assigned an intensity using 1 of 3 severity grades:

Mild: aware of the event, but easily tolerated

Moderate: enough discomfort to the patient to cause interference with usual activity

Severe: incapacitating – the patient is unable to work or perform usual activities

Additional statistical methods

The blocked Wilcoxon rank-sum test was also used as the primary method to evaluate treatment group differences for secondary and other efficacy endpoints classified as continuous measures or as counts and the proportion of time that a particular outcome occurred.

Time-to-event endpoints used the log-rank test to evaluate treatment differences. Cox's proportional hazards regression model was used to evaluate treatment effects in the presence of pertinent baseline covariates.

All statistical tests of treatment differences and related inferential summaries for the secondary and other efficacy endpoints were descriptive.

Additional preferred terms from Table 3

Injury, poisoning, and procedural complications includes post-procedural constipation, procedural nausea, procedural pain, ankle fracture, contusion, femur fracture, post-embolization syndrome, post-procedural bile leak, post-procedural complication, vascular pseudoaneurysm, wound secretion, and arthropod bite.

Investigations also includes alanine aminotransferase increase, aspartate aminotransferase increase, blood alkaline phosphatase increase, blood lactic acid increase, blood magnesium decrease, blood phosphorus decrease, abnormal blood pressure, body temperature increase, Eastern Cooperative Oncology Group performance status, echocardiogram, hematocrit decrease, hepatic enzyme increase, abnormal liver function test, red blood cell count decrease, anticoagulation drug level below therapeutic, blood albumin decrease, blood thyroid-stimulating hormone increase, blood urea increase, C-reactive protein increase, cardiac murmur, creatinine renal clearance decrease, diagnostic procedure, electrocardiogram QT interval prolonged, electrocardiogram ST-T change, international normalized ratio increase, neutrophil count increase, protein total decreased, and white blood cell count increased.

Musculoskeletal and connective tissue disorders also includes muscle spasms, musculoskeletal chest pain, pain in extremity, bone pain, arthritis, bursitis, coccydynia, and flank pain.

Psychiatric disorders also includes anxiety, apathy, decreased interest, insomnia, nervousness, sleep disorders, agitation, confusional state, and restlessness.
